# Multisystem Inflammatory Syndrome in Children in a 15-Year-Old Male with a Retropharyngeal Phlegmon

**DOI:** 10.1155/2020/6668371

**Published:** 2020-11-18

**Authors:** Paul Han, Jelena Douillard, Jerry Cheng, Anusha Ramanathan, David Tieu, Timothy Degner

**Affiliations:** ^1^Southern California Permanente Medical Group, Los Angeles, CA, USA; ^2^University of California Los Angeles, Los Angeles, CA, USA

## Abstract

This is a case of a 15-year-old male with an initial diagnosis of a retropharyngeal phlegmon who ultimately developed new symptoms and laboratory findings consistent with MIS-C. This case report demonstrates an atypical initial presentation for MIS-C that has not been reported in the literature.

During the novel coronavirus 2019 (COVID-19) pandemic, it has been established that children with primary infections with this virus are less severely affected and less likely to be hospitalized compared to adults [1–3]. However, citing evidence from both the United Kingdom and Italy [4, 5], an official health advisory was released by the Centers for Disease Control and Prevention (CDC) in May 2020 on what is now known as Multisystem Inflammatory Syndrome in Children (MIS-C) associated with COVID-19, as well as a case definition [6]. Since then, the American College of Rheumatology has published a diagnostic pathway for MIS-C, including clinical features (rash, gastrointestinal symptoms, edema of the hands or feet, oral mucosal changes, conjunctivitis, lymphadenopathy, and neurologic symptoms), as well as a tiered laboratory workup [7], and the American Academy of Pediatrics has provided interim guidance [8]. We are reporting on a case of a 15-year-old male who initially presented with symptoms consistent with and radiographic evidence of a retropharyngeal phlegmon who was subsequently diagnosed with MIS-C. 

A 15-year-old male with a history of epilepsy and asthma initially presented to the emergency department with two days of frontal headaches, sore throat, and fever to a maximum of 104 degrees Fahrenheit, as well as one day of neck pain and stiffness. His initial workup was significant for the following: white blood cell count 13,300/mcL, hemoglobin 14.9 g/dL, platelet count 202,000/mcL, absolute neutrophil count 10,860/mcL, absolute lymphocyte count 1,006/mcL, sodium 136 mEq/L, C-reactive protein (CRP) 117.2 mg/L, throat culture negative for Group A *Streptococci*, negative COVID-19 PCR, and neck CT scan with palatine tonsillar enlargement and a retropharyngeal fluid density extending down to C7/T1 (Figure 1). The patient was admitted for further care and was started on intravenous (IV) ampicillin-sulbactam and IV dexamethasone. Pediatric otolaryngology evaluated the patient and made the diagnosis of an early retropharyngeal phlegmon. The patient showed significant improvement on medical therapy, including IV antibiotics and dexamethasone, and he was discharged home on hospital day one with a course of oral amoxicillin-clavulanate.

One day after his discharge, the patient presented again to the emergency department with fever, headache, neck pain, shortness of breath, and midsternal chest pain, and during his evaluation, he had a brief syncopal episode. The patient's workup was significant for the following: white blood cell count 13,800/mcL, hemoglobin 12.8 g/dL, platelet count 230,000/mcL, absolute neutrophil count 11,660/mcL, absolute lymphocyte count 1,260/mcL, sodium 137 mEq/L, CRP 54.6 mL/L, ESR 19 mm/HR, negative COVID-19 PCR, troponin I 1.27 ng/mL, b-type natriuretic peptide (BNP) 204 pg/mL, and creatinine 0.62 mg/dL. CT angiogram of his chest did not reveal pulmonary emboli, and a repeat neck CT scan showed a decrease in the size of his retropharyngeal fluid density (Figure 2). The patient was then admitted for further care and was restarted on IV ampicillin-sulbactam, as well as IV clindamycin.

Soon after his second admission, he was found to have positive COVID-19 immunoglobulin G (IgG), and his echocardiogram showed mildly reduced ejection fraction without left ventricular dilatation, pericardial effusion, or coronary artery abnormalities [function was low normal by m-mode (27% fractional shortening and 53% ejection fraction), but by two-dimensional measurement, it was moderately reduced with 40% ejection fraction]. After discussion with pediatric rheumatology, there was concern for MIS-C, and he was given 1 g/kg intravenous immune globulin (IVIG) 5%, in addition to dexamethasone eight milligrams IV every eight hours. His mean arterial pressure also decreased to 55, and he was transferred to the pediatric intensive care unit (PICU). Additional workup during his second hospitalization did reveal the following: BNP increase to 591 pg/mL, absolute lymphocyte count decrease to 630/mcl, creatinine increase to 1.03 mg/dL, CRP increase to 102.8 mL/L, fibrinogen elevation to 577 mg/dL, D-dimer elevation to 3.13 mcg FEU/mL, ferritin elevation to 332 ng/mL, negative blood culture, and negative multiplex PCR respiratory panel. Pediatric hematology oncology was also consulted, and the patient was subsequently started on subcutaneous enoxaparin sodium. The patient did not require vasopressor or inotropic support in the PICU and was thus transferred to the general pediatric ward the next day with improved blood pressures and resolution of his fever and neck pain. The patient's troponin I, BNP, CRP, ferritin, fibrinogen, D-dimer, and absolute lymphocyte count all improved during his hospital stay, and he was discharged home on a dexamethasone wean, aspirin 81 milligrams orally daily, and a 14-day course of amoxicillin-clavulanate. After discharge, the patient's echocardiogram showed normal biventricular function with a left ventricular ejection fraction of 71 percent by m-mode. He required continued steroids after discharge and was started on anakinra by pediatric rheumatology due to evidence of ongoing cytokine storm, further supporting his diagnosis of MIS-C.

Our patient demonstrates an atypical initial presentation for MIS-C that has not been reported in the literature. Although there are reports of retropharyngeal involvement in cases of MIS-C and multisystem inflammatory syndrome in adults, those patients did not present with primary concerns for a deep neck infection [9, 10]. However, our patient had the initial diagnosis of isolated retropharyngeal phlegmon without suggestion of MIS-C, and only on his second admission, a little over a day after his discharge, did he meet the CDC case definition for MIS-C with progression of symptoms, including fevers, severe illness, multi-organ involvement, and positive COVID-19 serology [6].

This syndrome does share many clinical features with Kawasaki disease [6, 7], and there are reports of pre-COVID-19 pandemic Kawasaki disease being associated with deep neck space abnormalities, including retropharyngeal phlegmon [11–14]. However, the patients in those case reports had clinical features consistent with incomplete or classic Kawasaki disease, unlike our patient. So, while the treatments for these two conditions are similar, and while the patient did respond to treatment, MIS-C was still considered the prevailing diagnosis.

We also considered the possibility that this patient may have had the misfortune of two separate, unrelated conditions of MIS-C and retropharyngeal phlegmon. With the knowledge that 50% of retropharyngeal infections are thought to be associated with an antecedent upper respiratory tract infection [15], it is unclear if this patient had a primary COVID-19 infection leading to his deep neck findings. However, an antecedent upper respiratory infection was less likely without prior symptoms and with a negative COVID-19 PCR and a negative respiratory panel multiplex PCR. Also, while there is evidence that the incidence of retropharyngeal abscesses has increased in the last decade [12], these infections are still most common in younger children, typically in those less than six years of age [16]. A 15-year-old presenting with a retropharyngeal phlegmon is not necessarily a rare occurrence, but there were no other known risk factors, such as history of trauma or a procedure, to explain this finding.

This case report describes a COVID-19 serology-positive patient with MIS-C who presented initially with evidence of a retropharyngeal phlegmon and was later diagnosed with MIS-C. While it remains to be seen if other patients with MIS-C may present with the same initial symptoms, thus further strengthening the link between this disease and deep neck pathology, it is important for providers to be aware that the diagnosis of a retropharyngeal phlegmon does not exclude the possibility of MIS-C.

## Figures and Tables

**Figure 1:**
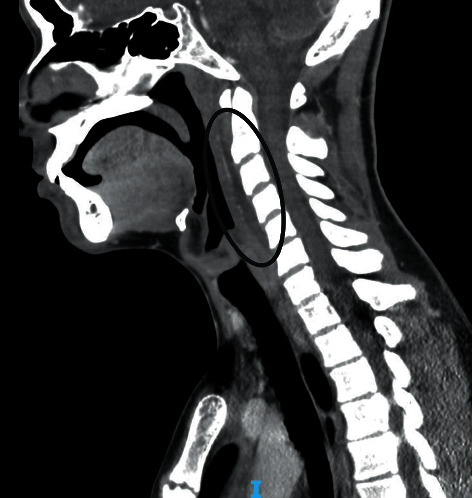
Palatine tonsillar enlargement and a retropharyngeal fluid density extending from the palatine tonsil down to C7/T1.

**Figure 2:**
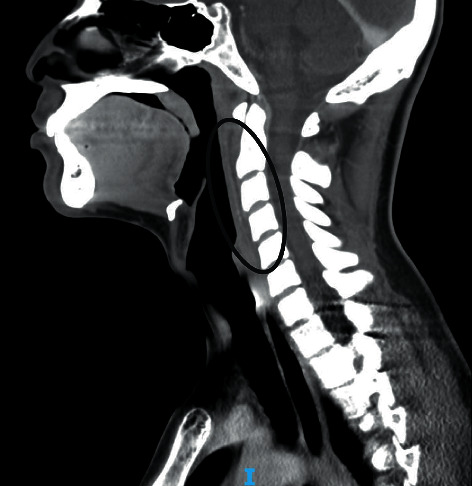
Decrease in the previously noted retropharyngeal phlegmon.

## Data Availability

No data were used to support this study.

## Abbreviations

COVID-19: Novel coronavirus 2019
CDC: Centers for Disease Control and Prevention
MIS-C: Multisystem inflammatory syndrome in children
CRP: C-reactive protein
IV: Intravenous
BNP: b-type natriuretic peptide
IgG: Immunoglobulin G
IVIG: Intravenous immune globulin
PICU: Pediatric intensive care unit.
